# Supplementary material for: Stable representation of a naturalistic movie emerges from episodic activity with gain variability
Source: Nat Commun. 2021 Aug 27;12:5170. doi: 10.1038/s41467-021-25437-2 (PMC8397750; doi:10.1038/s41467-021-25437-2)
Supplement: Supplementary file 1 — Supplementary Information [file 41467_2021_25437_MOESM1_ESM.pdf]

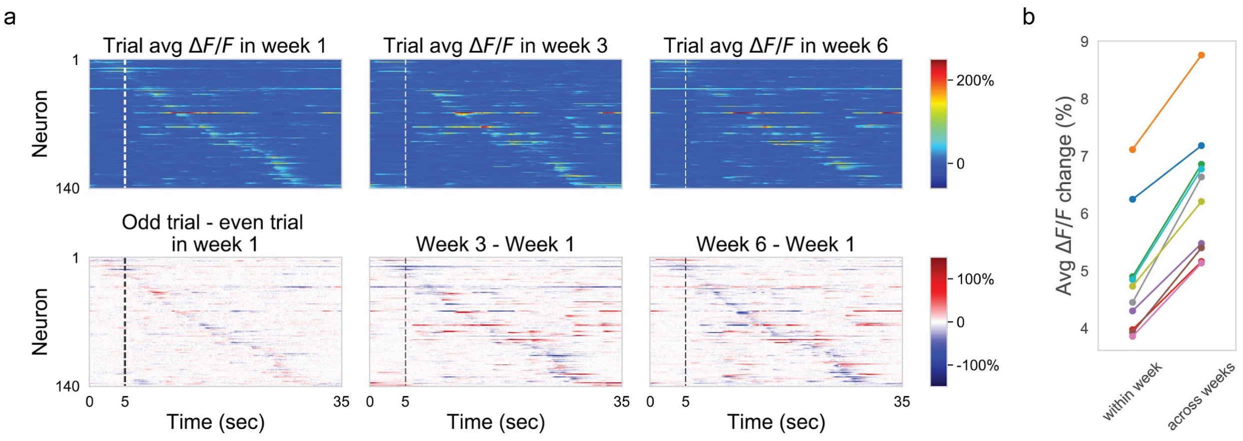

Supplementary Figure 1 Single neuron responses to natural movies are unstable across weeks.

a. Trial-averaged $\Delta F/F$ ordered by their peak time in week 1 are shown for different weeks for one example imaging field in the upper row. Difference between trial-averaged $\Delta F/F$ with the same neuron ordering are shown in the lower row.

b. $\Delta F/F$ change within week (L1 norm of difference between trial-averaged $\Delta F/F$ of even and odd trials) averaged across weeks is plotted against $\Delta F/F$ change across weeks (L1 norm of difference between trial-averaged $\Delta F/F$ of different weeks) averaged across all the week pairs (one-sided Wilcoxon signed-rank test, p = 0.0025, 10 imaging fields). Different colors denote different imaging fields.

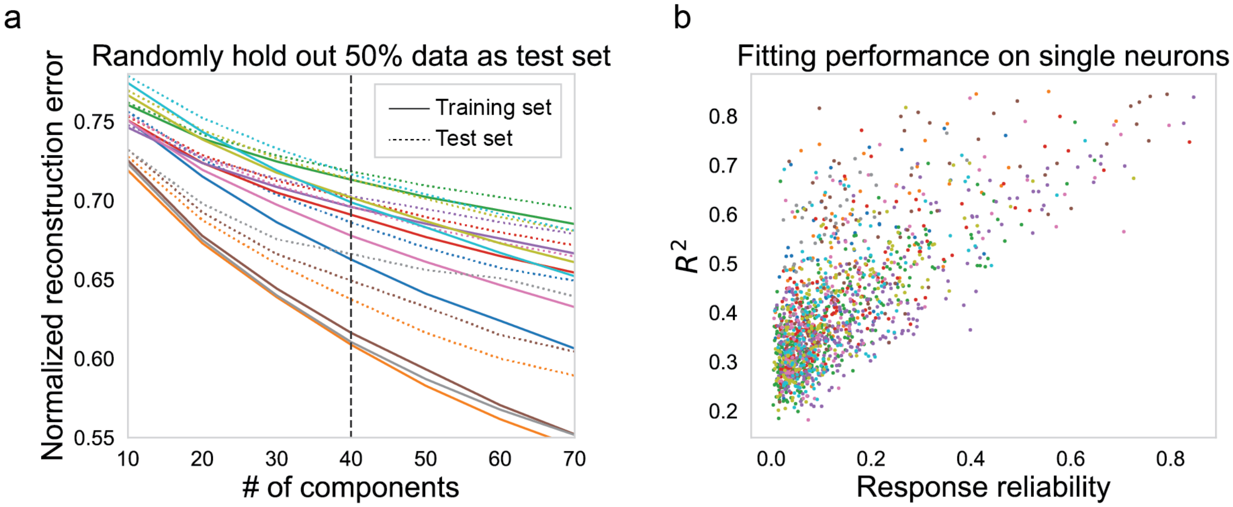

Supplementary Figure 2 Fitting performance of TCA.

a. Normalized reconstruction error plotted against the number of components of TCA for training set and test set for 10 imaging fields. Color denotes different imaging fields. Dashed line denotes the TCA model with 40 components.

b. Fitting performance R^2 plotted against response reliability for neurons pooled from 10 imaging fields. Each dot represents one neuron. Color denotes different imaging fields.

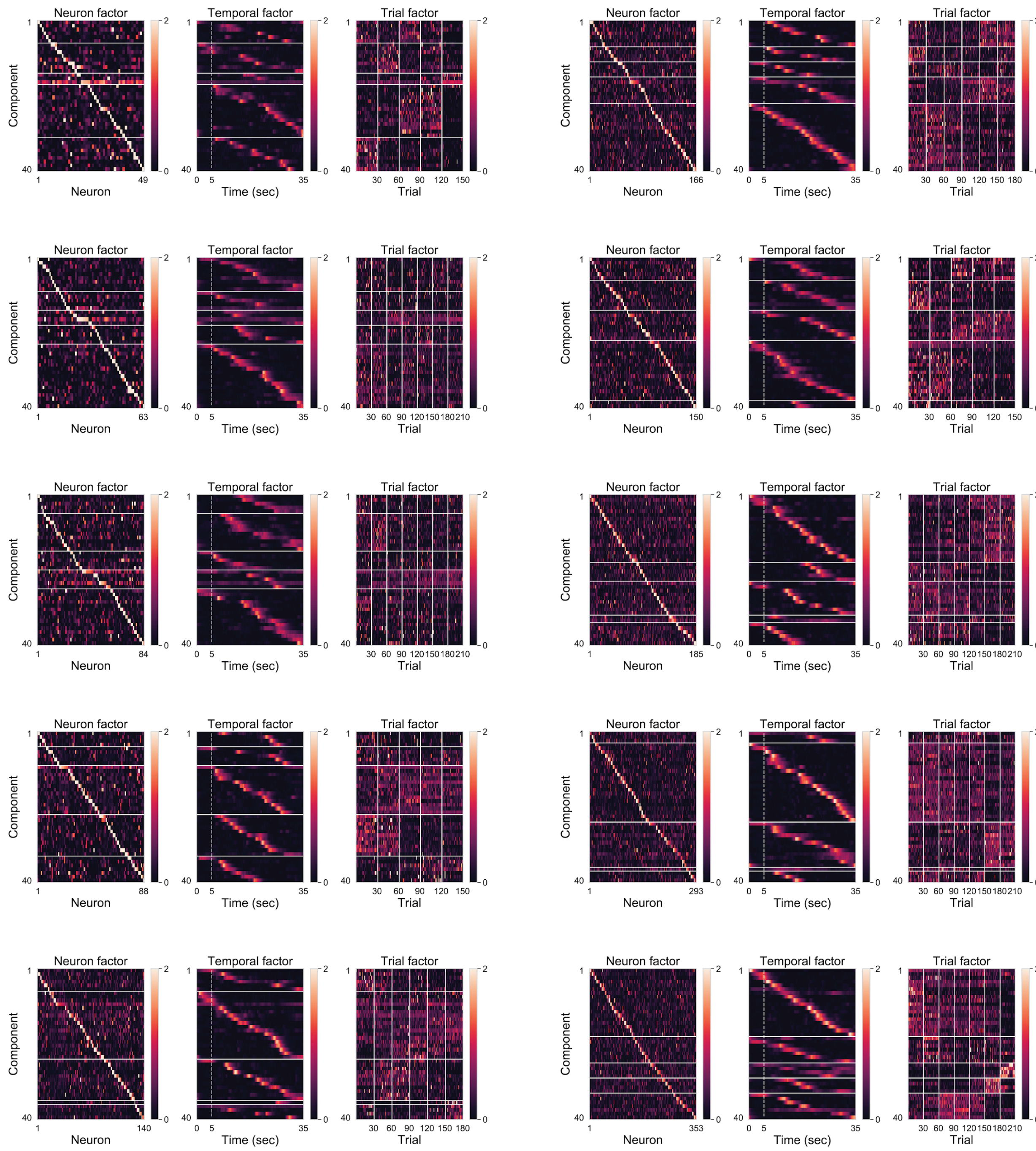

Supplementary Figure 3 Latent factors resembling episodic activity with gain changes capture the across-week variability.

Neuron, temporal, and trial factors of nonnegative TCA with 40 components for 10 imaging fields. We ordered neurons in the neuron factors by their dominant components. Colormap maximum values are set to 2. We ordered components according to the K-means clustering on their trial factors. Within each thus determined cluster, we further ordered the components by the time to peak in their temporal factors.

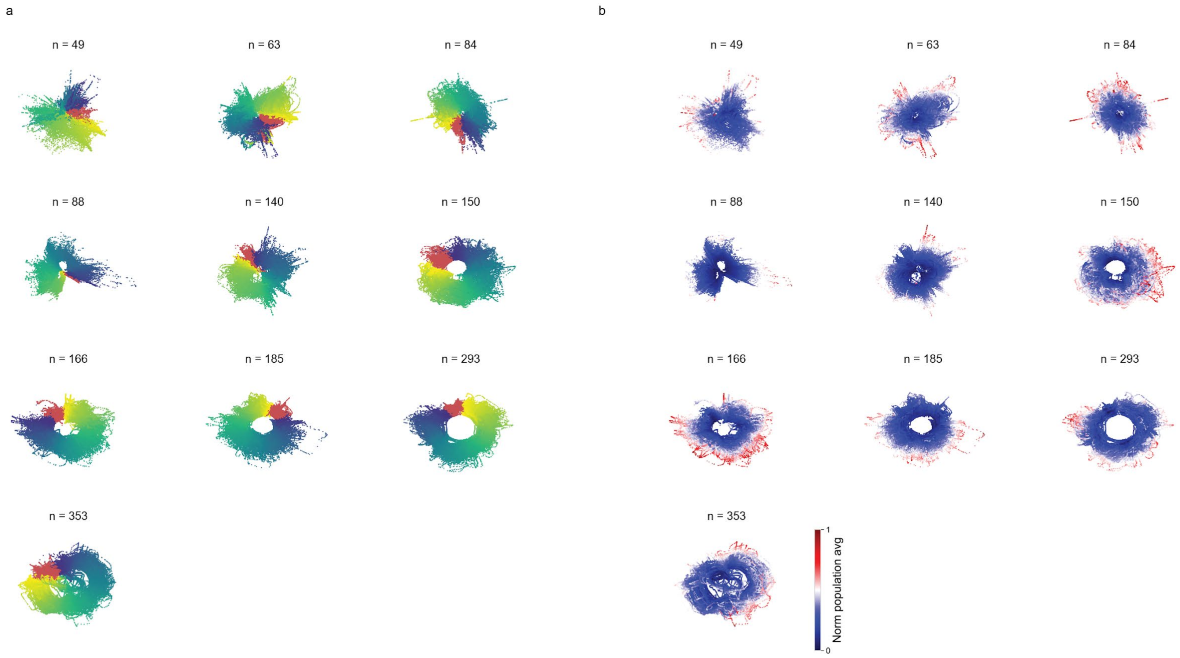

Supplementary Figure 4 Neural manifold for natural movie population responses in V1.

a. 2-dimensional neural manifold extracted from reconstructed (denoised) $\Delta F/F$ population activity across weeks using Isomap for 10 imaging fields. Each dot represents instantaneous population activity in the test set. Color of the dot (Same colormap as Fig. 4a) indicates the corresponding time in the trial.

b. Same neural manifold as in a. Color of the dot indicates the corresponding normalized reconstructed (denoised) $\Delta F/F$activity averaged over neurons in the imaging field.

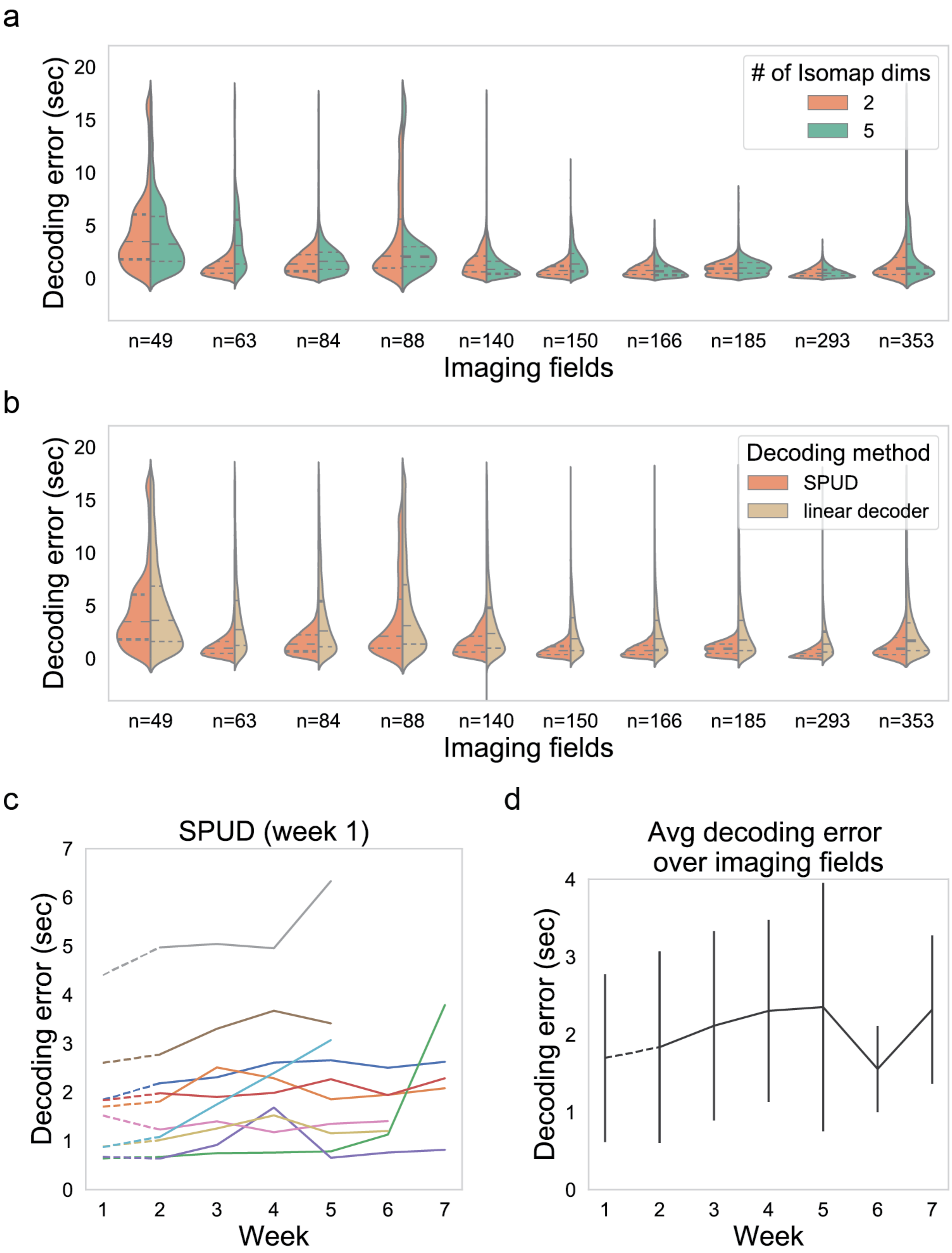

Supplementary Figure 5 Decoding performance of SPUD.

a. Decoding error (absolute circular difference between decoded time and actual time) of SPUD on 2-dimensional neural manifold plotted against decoding error of SPUD on 5-dimensional neural manifold for all the imaging fields. Imaging fields were ordered by the number of recorded neurons.
b. Decoding error (absolute circular difference between decoded time and actual time) of SPUD on 2-dimensional neural manifold plotted against decoding error of linear decoder using reconstructed (denoised) $\Delta F/F$ population activity for all the imaging fields. Imaging fields were ordered by the number of recorded neurons. Decoding error of SPUD was significantly smaller than decoding error of linear decoder for all the imaging fields (one-sided Mann-Whitney U test, 14700 timepoints for imaging field n = 63, 84, 185, 293, 353; 12600 timepoints for imaging field n = 140, 166; 10500 timepoints for imaging field n = 49, 88, 150, p-values for different imaging fields are 0.0011 (n = 49), 4.15×10^{-78} (n = 88), 0.0 for the other imaging fields)
c. Decoding errors of SPUD trained on data from odd trials in week 1 and tested on data from even trials in week 1 and trials from other weeks. Different colors correspond to different imaging fields.
d. Decoding errors averaged over imaging fields for SPUD trained on data from odd trials in week 1. Error bar denotes the standard deviation over imaging fields (10 imaging fields for week 1-5; 7 imaging fields for week 6; 5 imaging fields for week 7).The decoding errors pooled from later weeks were not significantly different from the decoding errors for week 1 across imaging fields (p = 0.11, two-sided Mann-Whitney U test, 10 experiments for week 1, 52 experiments pooled for later weeks).

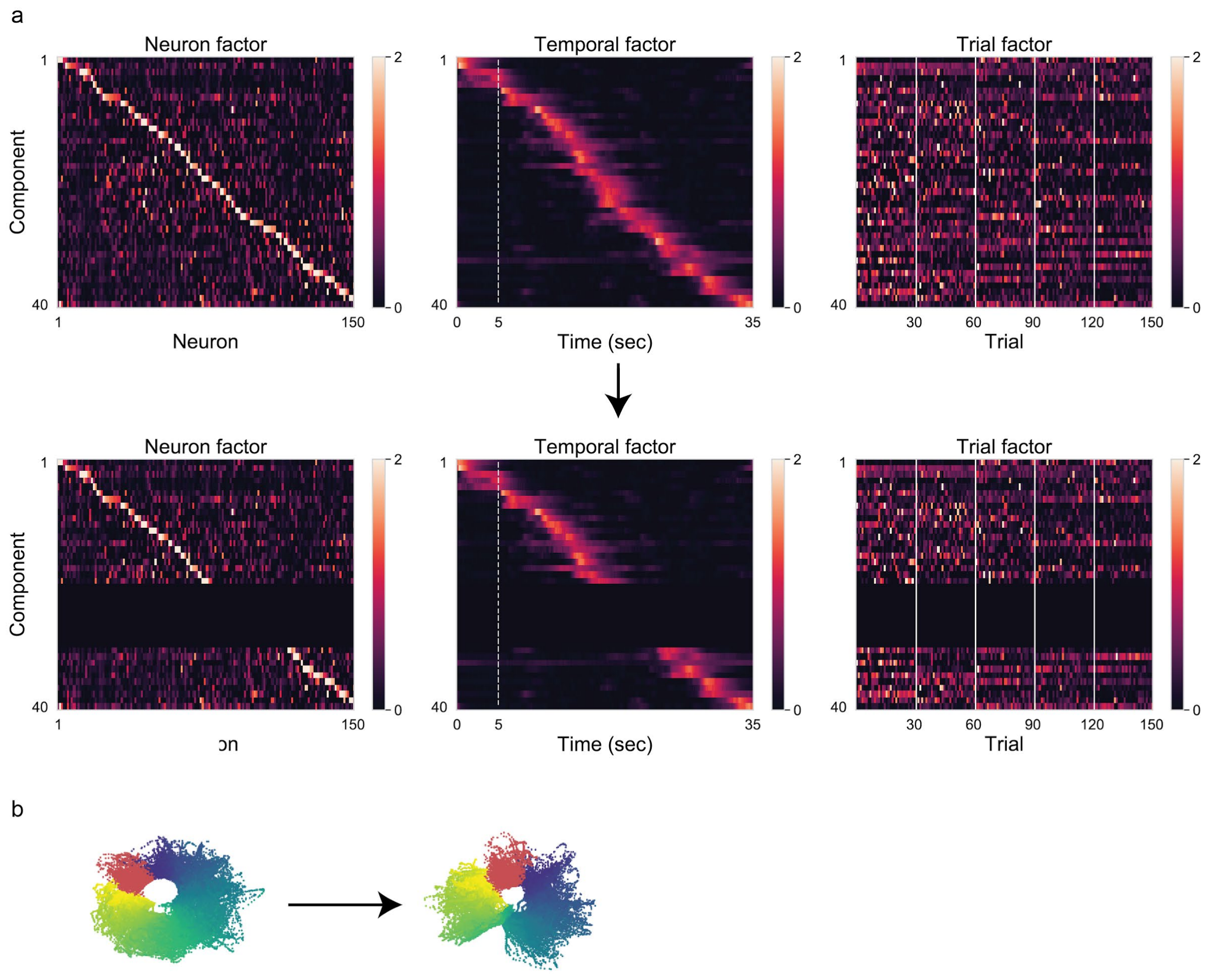

Supplementary Figure 6 Removing episodic activity during a certain time window leads to collapse of the ring manifold.

a. TCA components of one imaging field (n = 150) and the modified TCA components. For modified TCA components, we set the components with episodic activity during a certain time window to zeros.
b. 2-dimensional neural manifold extracted from reconstructed $\Delta F/F$ population activity from original TCA components and neural manifold extracted from reconstructed $\Delta F/F$ population activity from modified TCA components.

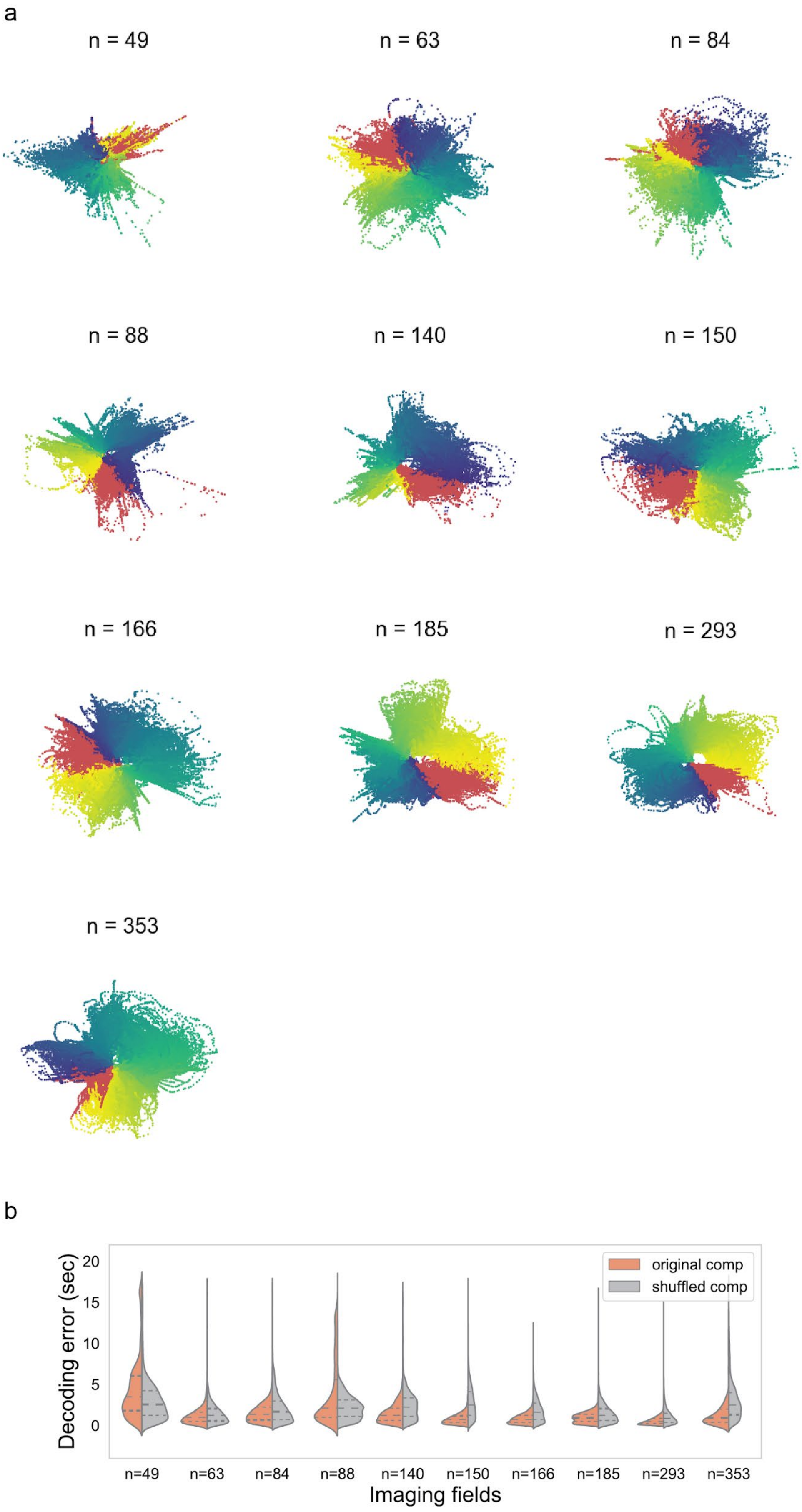
a
n = 49
n = 63
n = 84
n = 88
n = 140
n = 150
n = 166
n = 185
n = 293
n = 353
b
Decoding error (sec)
20
15
10
5
0
original comp
shuffled comp
n=49
n=63
n=84
n=88
n=140
n=150
n=166
n=185
n=293
n=353
Imaging fields

Supplementary Figure 7 Neural manifold of reconstructed $\Delta F/F$ population activity from TCA components with shuffled factors

a. 2-dimensional neural manifold extracted from reconstructed $\Delta F/F$ population activity from TCA components with shuffled factors as described in Fig. 7c using Isomap for 10 imaging fields. Each dot represents instantaneous population activity in the test set. Color of the dot (Same colormap as Fig. 4a) indicates the corresponding time in the trial.
b. Decoding error (absolute circular difference between decoded time and actual time) of SPUD on 2-dimensional neural manifold from reconstructed $\Delta F/F$ population activity from original TCA components plotted against decoding error of SPUD on 2-dimensional neural manifold from reconstructed $\Delta F/F$ population activity from TCA components with shuffled factors as described in Fig. 7c for all the imaging fields. Imaging fields were ordered by the number of recorded neurons. Except for 2 imaging fields (n = 49, n = 88), decoding error of reconstructed $\Delta F/F$ population activity from original TCA components was significantly smaller than decoding error of reconstructed $\Delta F/F$ population activity from TCA components with shuffled factors (one-sided Mann-Whitney U test, 14700 timepoints for imaging field n = 63, 84, 185, 293, 353; 12600 timepoints for imaging field n = 140, 166; 10500 timepoints for imaging field n = 49, 88, 150, p-values for different imaging fields are 1.0 (n = 49, n = 88), 8.62×10^{-92} (n = 63), 4.60×10^{-105} (n = 84), 6.45×10^{-257} (n = 185), 0.0 for the other imaging fields). Note that for TCA components with shuffled factors, we chose ten trial-averaged projected instantaneous population activity evenly distributed in time as the initial knots for the SPUD to enable fair quantitative comparison (see Methods).

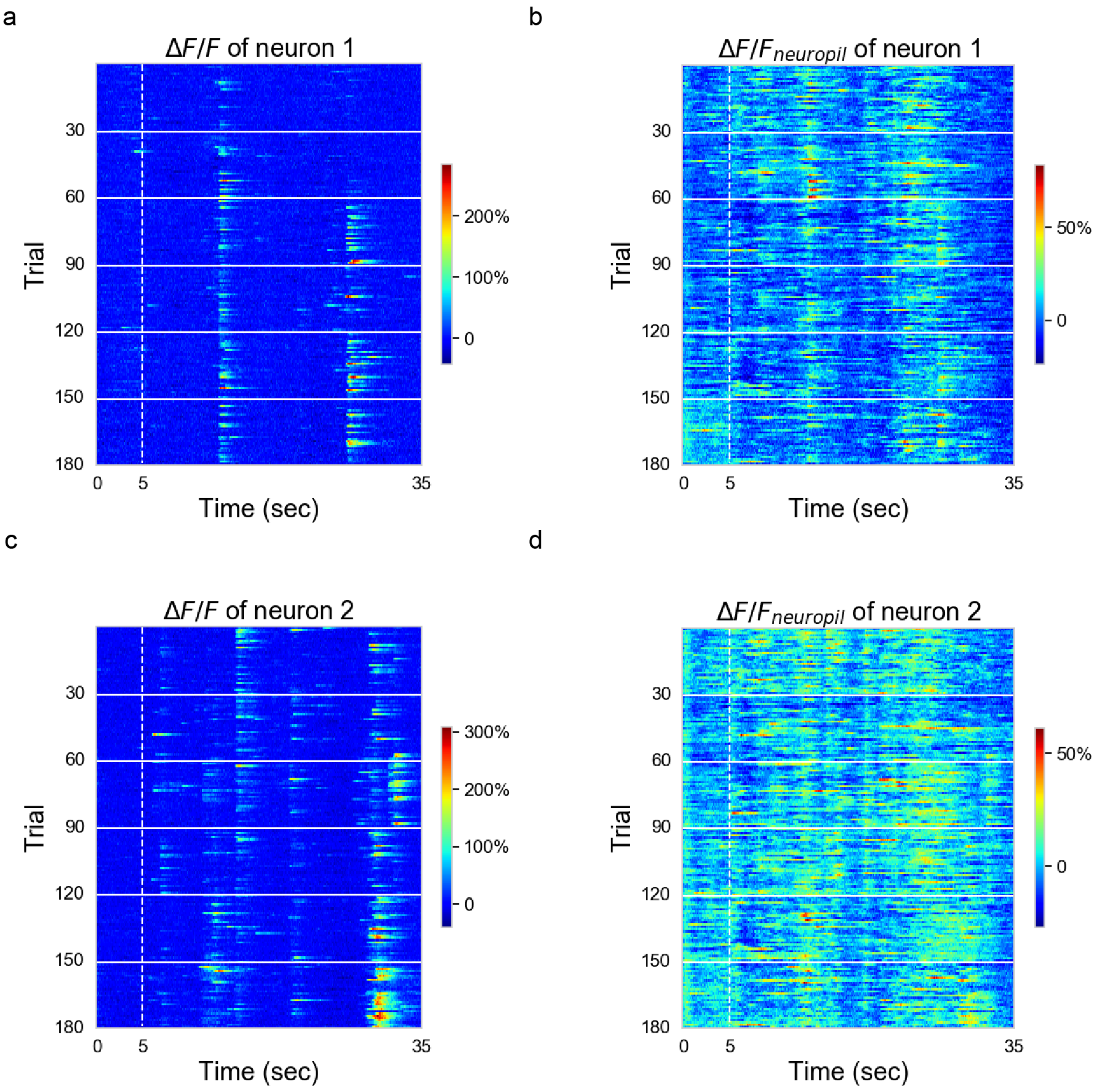

Supplementary Figure 8 Example neuropil signals across weeks.

a. $\Delta F/F$ of one example neuron, neuron 1.
b. $\Delta F/F_{neuropil}$ of neuron 1.
c. $\Delta F/F$ of another example neuron, neuron 2.
d. $\Delta F/F_{neuropil}$ of neuron 2.
