# Supplementary material for: Stable representation of a naturalistic movie emerges from episodic activity with gain variability
Source: Nat Commun. 2021 Aug 27;12:5170. doi: 10.1038/s41467-021-25437-2 (PMC8397750; doi:10.1038/s41467-021-25437-2)
Supplement: Supplementary file 3 — Description of Additional Supplementary Files [file 41467_2021_25437_MOESM3_ESM.pdf]

Description of Additional Supplementary Files

Supplementary Software: This zip file contains a modified version of the SPUD code (the original SPUD code is provided at https://fietelab.mit.edu/code/), an example dataset and a Jupyter notebook to reproduce some of the main figures.
